# Soluble expression and purification of Bluetongue Virus Type 1 (BTV1) structure protein VP2 in *Escherichia coli* and its immunogenicity in mice

**DOI:** 10.7717/peerj.10543

**Published:** 2021-01-04

**Authors:** Aiping Wang, Jiajia Yin, Jingming Zhou, Hongfang Ma, Yumei Chen, Hongliang Liu, Yanhua Qi, Chao Liang, Yankai Liu, Jinge Li, Gaiping Zhang

**Affiliations:** Zhengzhou University, School of Life Sciences, Zhengzhou University, Henan, PR China

**Keywords:** Bluetongue virus, Recombinant protein VP2, Solubility, Immunogenicity

## Abstract

**Background:**

The VP2 on the surface of the virus particle is the main structural protein of BTV, which can induce the host to produce neutralizing antibodies and play an important role in the antiviral immunity process. This study aimed to obtain the soluble VP2 and analyze its immunogenicity.

**Methods:**

The gene encoding the full-length VP2 of BTV1 was amplified by PCR. The products from restriction enzyme digestion and ligase reaction between VP2 and vector pET-28a were transformed into *E.coli* DH5α. After PCR and sequencing detection, the positive plasmid PET28a-VP2 was transformed into *E.coli* BL21(DE3) and Rosetta(DE3) competent cells, expression induced by IPTG. The fusion protein was expressed in the optimized conditions with the induction of IPTG, purified by affinity chromatography and identified by SDS-PAGE and Western blotting. A total of 5 Balb/c mice aged 6–8 weeks were immunized with the fusion protein at a dose of 30 µg per mouse. Each mouse was immunized three times at an interval of 3 weeks.

**Results:**

The recombinant plasmid PET28a-VP2 was successfully constructed. The expression strains were induced by 0.4 mmol/L IPTG at 16 °C for 10 h, and BTV1 VP2 was expressed in a soluble form. The purity of the recombinant VP2 protein (∼109 kDa) was about 90% in the concentration at 0.2 mg/ml afterpurification. The purified VP2 had good immunoreactivity with BTV1 positive serum. Taken together, thisstudy offered a route for producing soluble BTV VP2, which retains activity and immunogenicity, to bebeneficial to the research on developing BTV vaccine, and lay the foundation for further research on BTV.

## Introduction

Bluetongue (BT) is a severe noncontagious infectious disease, which is common in sheep and wild ruminants, and occasionally occurs in cattle, camels and carnivores (Sohail et al., 2018). Infected animals often develop symptoms of fever, cyanotic tongue, ulcers in the mouth and stomach and even death (Thiry et al., 2008). The first detailed descriptions of BT in sheep were in 1905, nowadays, BT is listed as a notifiable animal infectious disease by OIE and occurs throughout the world causing significant economic losses (Sperlova & Zendulkova, 2011; OIE, 2019).

Bluetongue virus (BTV) is the pathogen of BT and a member of the family *Reoviridae*, genus *Orbivirus* (Huismans & Erasmus, 1981). The virus particle has no envelope, icosahedral symmetry about 90 nm in diameter containing a core particle with maximum diameter around 73 mm Up to now, 27 serotypes of BTV are recognized with no cross-protection between each serotype (Maan Narender et al., 2012). The BTV linear dsRNA genome is approximately 19 kb consisting of 10 segments (Seg 1 to Seg 10) encoding seven structural proteins (VP1-VP7) and four nonstructural proteins (NS1, NS2, NS3/NS3a, NS4). The double capsid structure is formed by four structural proteins, i.e., VP2, VP5, VP3, and VP7. Especially, VP2, encoded by the L2 gene of BTV, is the main structural protein located on the surface of virions (Schwartz-Cornil et al., 2008). Moreover, VP2 is responsible for virus attachment and haemagglutination, inducing neutralizing antibodies and viral serotype determination (Bhattacharya, Noad & Roy, 2007). Hence, the structural protein VP2 with multiple epitopes, good specificity and high sensitivity was selected as a good antigen.

Currently, several genetic engineering BT vaccines including vaccines based on BTV proteins and viral vector vaccines are under development and have an extensive prospect. The serotype-specific immunodominant VP2 protein was selected as the antigen of the BTV protein vaccine. Protein has been studied in bacteria (Xie et al., 2018), in insect cells (Delos et al., 1993; Inumaru & Roy, 1987; Roy, French & Erasmus, 1992; Urakawa et al., 1994), in yeast (Athmaram et al., 2007), and in plants (Fay et al., 2019). Moreover, the prokaryotic expression is most rapid, money-saving and easy to operate, although most of proteins expressed in Prokaryotic system are insoluble and inactive. Mohd Jaafar et al. (2014) successfully expressed two domains (aa 63-471and 555-956) of BTV4 VP2 in soluble form in bacteria. Li et al. (2016) induced the expression of soluble BTV25 VP2 protein with maltose tag in *E.coli*. Zhou et al. (2017) obtained three truncated BTV4 VP2 proteins using Prokaryotic expression system. However, no scholars have expressed the full-length BTV1 VP2 in prokaryotic.

Therefore, we decided to express and purify soluble BTV1 VP2 in the Prokaryotic ** expression system and analyzed its immunogenicity in mice. We expect to lay the foundation for further study on developing potential and safe subunit vaccine for prevention and control BT effectively.

## Materials & Methods

### Ethics statement

Animal experiments were performed in accordance with the guidelines of the Chinese Council on Animal Care. The research protocol was approved by the Animal Care and Use Committee at School of life Sciences, Zhengzhou University.

### Experimental animals

Ten adult male albino mice, which were chosen to avoid data interference from hormonal variations, were purchased in September 2019 from the Animal Experimental Center of Zhengzhou University. The mice were kept in a room maintained at a temperature of 25 ± 2 °C with a 12-h light/dark cycle, and they had free access to food and water. The experimental environment meets the requirements of GB-14925. This study was performed in strict compliance with standard recommendations in the Guide for the Management and Use of Laboratory Animals (published in May 2016). This protocol was approved by the Joint Unit of Zhengzhou University-the Animal Ethics Review Committee of the Key Laboratory of Animal Immunology, Henan Academy of Agricultural Sciences (LLSC100166). In order to avoid the impact of immunogen on the physical and mental health of mice by the experiment, they were sacrificed by cervical dislocation method under xanthazine anesthesia to reduce the pain of the mice.

### Establishment of recombination expression plasmid

A pair of BTV1 VP2 primers containing *EcoR*I and *Xho*I sites were designed according to the complete gene sequence of BTV1 VP2 (GenBank accession number KF664124) and used to amplify the full length VP2 gene (Synthesized by Sangon Biotech, China) The sense primer was 5′-ATGGACGAGCTGGGTAT-3′ and the antisense primer was 5′-TTAAACGTTGAGGAGCTTAGT-3′. The PCR amplification was carried out using PrimeSTAR DNA polymerase kits (TaKaRa, China) with the following reaction conditions: a predenaturation at 98 °C for 10 sec, 32 cycles of denaturation at 98 °C for 10 s, annealing at 55 °C for 30 s, and extension at 68 °C for 30 s; and an extension at 68 °C for 5 min. The PCR product was visualised in a 1% agarose gel and then purified using DNA Gel Extraction Kit (OMEGA, US). Then, the extracted VP2 gene was inserted between the *EcoR* I and *Xho* I sites of the vector pET-28a. The recombinant vector pET-28a-VP2 was identified by restriction enzyme digestion and the BTV1 VP2 insert was verified by sequencing. The recombinant expression vector was confirmed by restriction enzyme digestion and sequencing. *E.coli* DH5α was used for amplification of the recombinant plasmid. Then, pET28a-VP2 was transformed into *E.coli* BL21(DE3) and *E.coli* Rosetta(DE3) competent cell lines to induce the expression of the His-tagged BTV1 VP2 protein.

### Expression and solubility analysis of recombinant protein VP2

Single colonies of *E.coli* BL21 (DE3) and *E.coli* Rosetta (DE3) transformed with the recombinant expression plasmid pET28a-VP2 were respectively inoculated in the tube containing 5 ml of LB medium (containing 50 µg/mL of kanamycin, 50 µg/mL of kanamycin and 34 µg/mL of chloramphenicol) and cultivated overnight at 37 °C in a shaking incubator. Until the OD_600_ of the culture was about 0.6–0.8, the expression of VP2 was induced by the addition of isopropyl β-D-thiogalactoside (IPTG). After induction, the expression of the fusion protein was analyzed by SDS-PAGE, and the cells were harvested at 25 °C by centrifugation at 10,000 rpm for 15 min. The supernatant was discarded and the cell pellet was washed, frozen, resuspended in phosphate-buffered saline (PBS, pH 7.4), and then disrupted by sonication. The eluted fractions were analyzed by 10% SDS-PAGE. The results of SDS-PAGE were obtained using a Universal Hood III gel imaging system (Biorad, US).

### Optimal expression conditions of recombinant protein VP2

The results of the expression of VP2 in the two expression strains were analyzed,VP2 protein in Rosetta (DE3) strain mainly existed in soluble form. Then, the Rosetta (DE3) strain was selected to express the target protein VP2. Moreover, the expression conditions for pET28a-VP2 (Rosseta) containing the temperature, IPTG concentration and time were explored. Recombinant protein VP2 was expressed in a series of inducting temperatures including 16 °C, 25 °C, 30 °C and 37 °C. The fusion protein was induced by a group of IPTG at varied concentrations containing 0.2 mmol/L, 0.4 mmol/L, 0.6 mmol/L, 0.8 mmol/L and 1.0 mmol/L. Afterwards, the optimal expression time was determined according to the arithmetic progression including five values increasing from the beginning value 0 h to the ending value 10 h, using the optimal temperature and IPTG concentration. All results are analyzed using SDS-PAGE.

### Purification of recombinant protein VP2

The recombinant protein VP2 was purified by the Ni-NTA agarose (Qiagen, Hilden, Germany). Briefly, the bacterium was resuspended with binding buffer (20 mM of Tris-HCl, 300 mM of NaCl, pH 8.2) and treated by ultrasonic, then, the supernatant was obtained by centrifugation at 12,000 rpm for 10 min. Then the supernatant was added to a pre-equilibrated Ni-chelating affinity chromatography.Then the mixture was incubated at 4 °C at 200 rpm for more than 2 h. After incubation, the wash buffer (20 mM of Tris-HCl, 300 mM of NaCl, 50 mM of imidazole, pH 8.2) was used to remove certain impurities. Then the recombinant protein VP2 attached onto Ni-chelating affinity chromatography was eluted by elution buffer (20 mM of Tris-HCl,300 mM of NaCl,100 mM of imidazole, pH 8.2). The purified protein was further analyzed using SDS-PAGE and Western blotting. For Western blotting, a HRP-conjugated 6 ×H is was used as the antibody.

#### Mass spectrometric identification of VP2 protein

It was further confirmed by mass spectrometry that the obtained protein was BTV1 VP2. The specific steps are as follows: The gel band containing the purified protein was carefully excised and transferred into a clean microfuge tube. Then, the sample was sent to biotechnology (Sangon, China) for mass spectrometry..

### Reactivity of recombinant protein VP2 with BTV1 positive serum

The reactivity of recombinant protein VP2 with positive serum (BTV1 positive serum obtained from mice immunized with BTV1 inactivated virus) was assayed by western blotting. BTV1 positive serum and HRP-conjugated goat anti-mouse monoclonal antibody were used as primary antibody and secondary antibody, respectively.

### Animal tests

For tested serotype, ten Balb/c mice at 6 ∼8 weeks old were randomly divided into two groups with five of each. The first group were immunized with recombinant VP2 at a dose of 30 µg/head as the experimental group. In the other group, the animals were not immunized as the control group. The Freund’s complete adjuvant mixed with recombinant VP2 was used in the first injection and the Freund’s incomplete adjuvant was used for the second and third immunizations. The interval time of immunization was 3 weeks, and blood was collected from the tail vein on 0, 7, 14, 21, 28, 35, 42, 49, 56 days after the first immunization. The serum of immunized mice was collected after centrifugation at 4000 rpm for 10 min.

### Evaluation of the immune effect

The immune effect of fusion protein VP2 was analyzed by dot-ELISA and indirect ELISA assay. The nitrocellulose filter membrane was coated by recombinant protein VP2 and inactivated BTV. The 200-fold diluted positive serum and 1000-fold diluted HRP-conjugated goat anti-mouse IgG antibody (Proteintech, US) were used as the primary antibody and the secondary antibody, respectively. The microplate was coated with 50 µl purified recombinant protein VP2 (0.05 mg/well) and primary antibodies was a series of gradient diluted serum beginning with 100-fold serum as well as the secondary antibody was HRP-conjugated goat anti-mouse IgG antibody. Moreover, the microplate was coated with 50 µl purified recombinant protein VP2 (0.05 mg/well) and a series of serum collected from the first week to the eighth week after immunization, as well as the secondary antibody was HRP-conjugated goat anti-mouse IgG antibody. The result of indirect ELISA was read using a Microplate Reader (BioTek Instruments, Inc, Winooski, VT, US) at 490 nm.

## Result

### Prokaryotic expression vector construction

**Figure 1:**
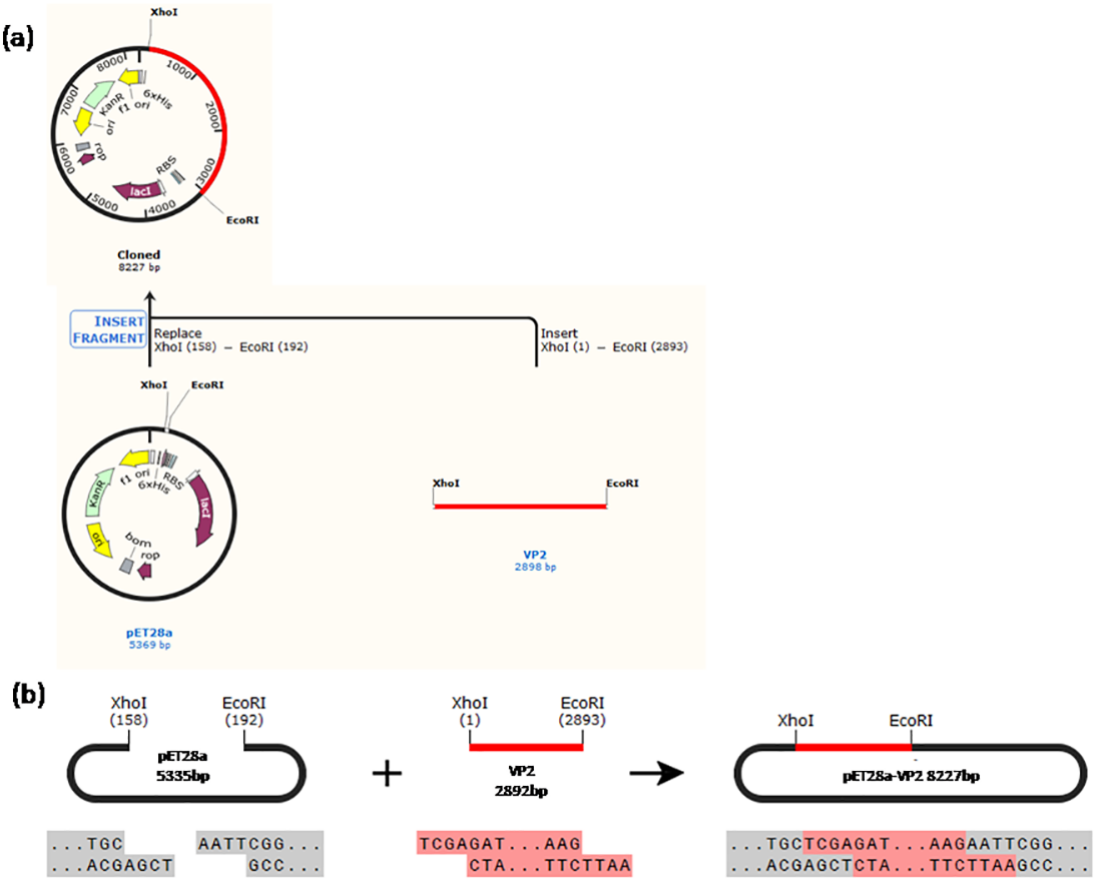
Schematic diagram of the constructed recombinant plasmid. (A) A history of construction to recombinate expression plasmid pET28a-VP2, (B) the sites and sequences of restriction enzyme *Xho*I and *EcoR*I.

**Figure 2:**
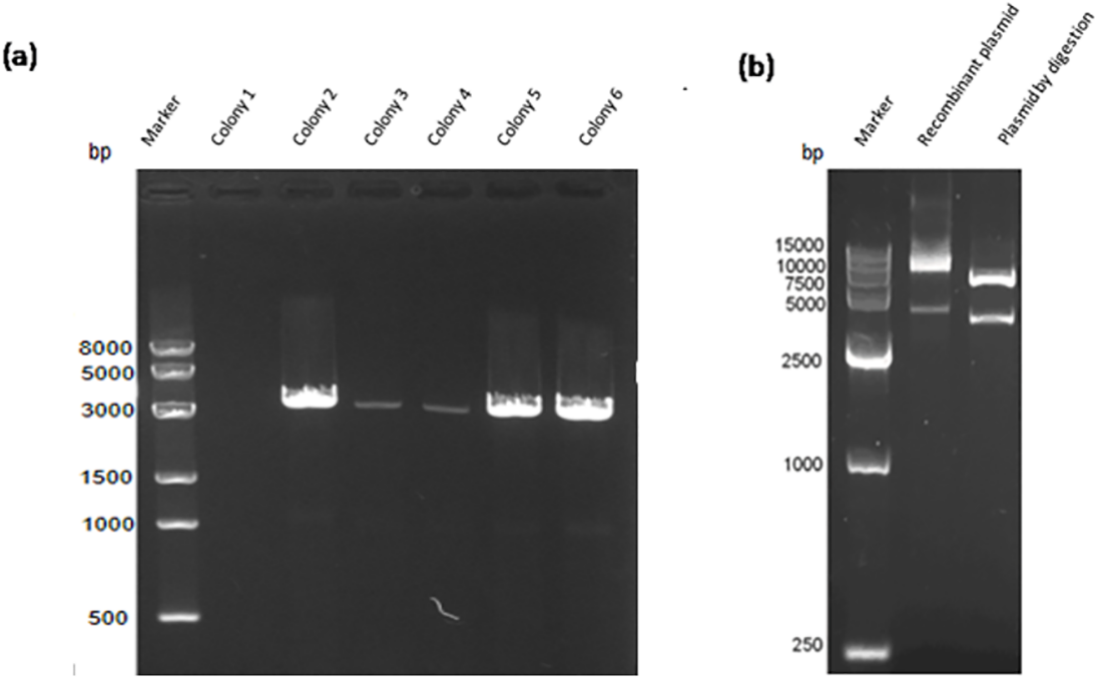
PCR detection and restriction enzyme digestion. (A) PCR result of bacterial liquid. Lane 1, marker. Lanes 2–7 denote selected 6 monoclonal colonies, clones 2–6 have specific bands at about 3,000 bp. (B) The recombinant expression vector pET-28a-VP2 digestion with restriction enzymes *EcoR*I and *Xho*I. Lane 1, marker. Lane 2 denotes pET-28a-VP2 plasmid and lane 3 denotes products of pET-28a-VP2 by restriction enzyme digestion.

Construction of the recombinant plasmid was in accordance with the principles of plasmid restructuring (Fig. 1). The prokaryotic expression vector pET28a-VP2 was successfully constructed by amplification of the target gene, restriction enzyme digestion, transforming into competent cells, coating plates, selecting monoclonal colony. The results of PCR detection showed that some monoclonal colonies had specific band was about 3000 bp in 1% agarose gel electrophoresis (Fig. 2A), which was accordant to the theoretical length of VP2 gene. The recombinant plasmid was digested by the restriction enzymes *EcoR* I and *Xho* I. Agarose gel electrophoresis of the digest revealed a DNA band at about 3000 bp (Fig. 2B). The inserted fragment was verified by sequencing, which was identical to the published BTV1 VP2 gene sequence.

### Expression and solubility identification of recombinant protein VP2

**Figure 3:**
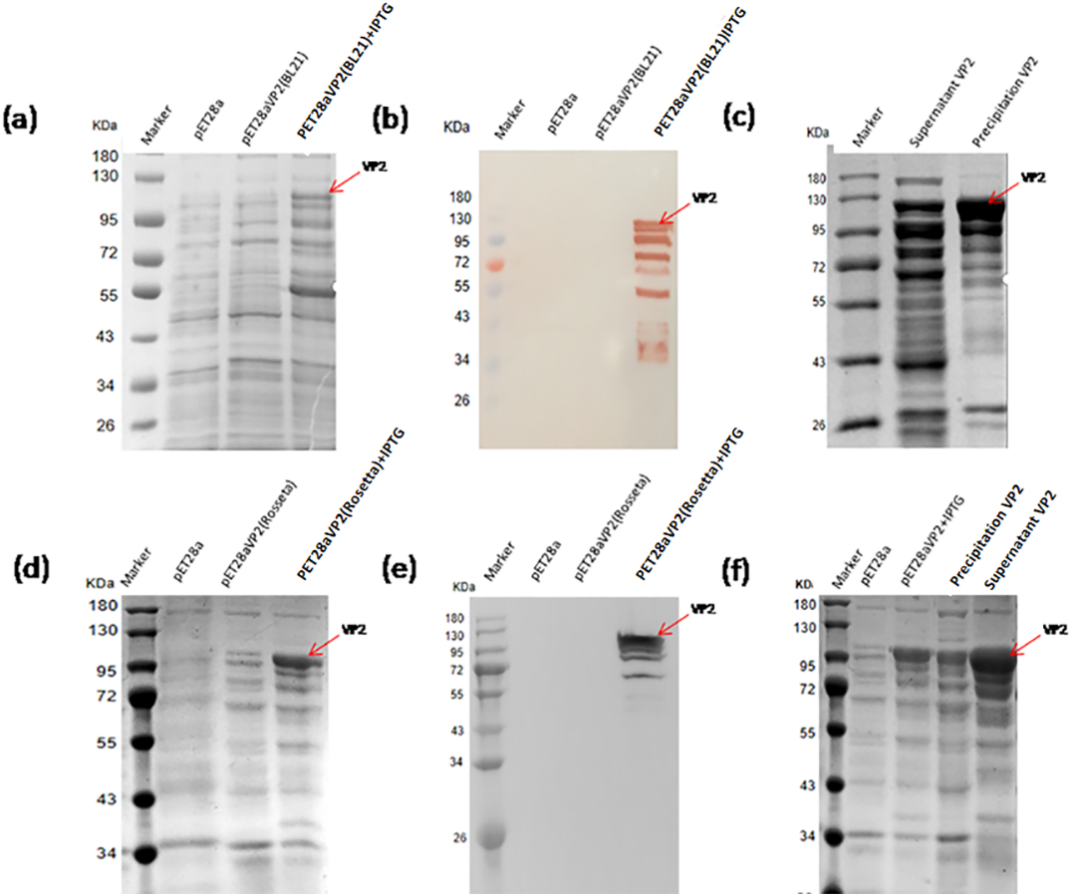
Expression of the fusion proteins in* E.coli* BL21(DE3) and Rosetta(DE3). SDS-PAGE analysis of the expression of recombinant VP2 in the BL21(DE3). (B) Western blotting analysis of the expression of recombinant VP2 by His-tag monoclonal antibody. Lane 1 denotes the middle-ranged protein marker. When the recombination strain was induced for 18 h by IPTG at 20 °C, 20 µL of the culture supernatant was collected. The culture supernatant mixed with the 5 ×loading buffer were boiled and then determined by SDS-PAGE as shown in lane 4 with target-free bacteria (lane 2) and the un-induced culture supernatant (lane 3) as control; Western blotting identification, the sample loaded in the lane was the same as SDS-PAGE. (C) The induced cells were cracked by ultrasonic, the lysate supernatant (lane 2) and the lysate pellet (lane 3) were analyzed by SDS-PAGE. (D) SDS-PAGE analysis of the expression of recombinant VP2 in the Rosetta(DE3). (E) Western blotting analysis of the expression of recombinant VP2 in the Rosetta(DE3) by His-tag monoclonal antibody. Lane 1 denotes the middle-ranged protein marker. The culture supernatant was determined by SDS-PAGE as shown in lane 4 with target-free bacteria (lane 2) and the un-induced culture supernatant (lane 3) as control. Western blotting identification, the sample loaded in the lane is the same as SDS-PAGE. (F) The solubility of VP2 in Rosetta(DE3) was analyzed by SDS-PAGE. The induced cells (lane 3) were cracked by ultrasonic, the lysate supernatant (lane 5) and the lysate pellet (lane 4) were identified by PAGE. In which, with target-free bacteria (lane 2) as control.

The pET28a-VP2 transformed into the strain BL21(DE3) and Rosetta(DE3) were induced by IPTG to express VP2. The results of SDS-PAGE and western blotting showed that the recombinant protein VP2 induced by IPTG was expressed (∼109 kDa) in both BL21(DE3) (Figs. 3A and 3B) and Rosetta (DE3) (Figs. 3D and 3E) strain. The size was consistent with the expected size. The solubility analysis of recombinant protein VP2 by SDS-PAGE showed that the supernatant expression of VP2 in Rosetta (DE3) was higher (Figs. 3C and 3F).

### Optimization of recombinant protein VP2 expression conditions

**Figure 4:**
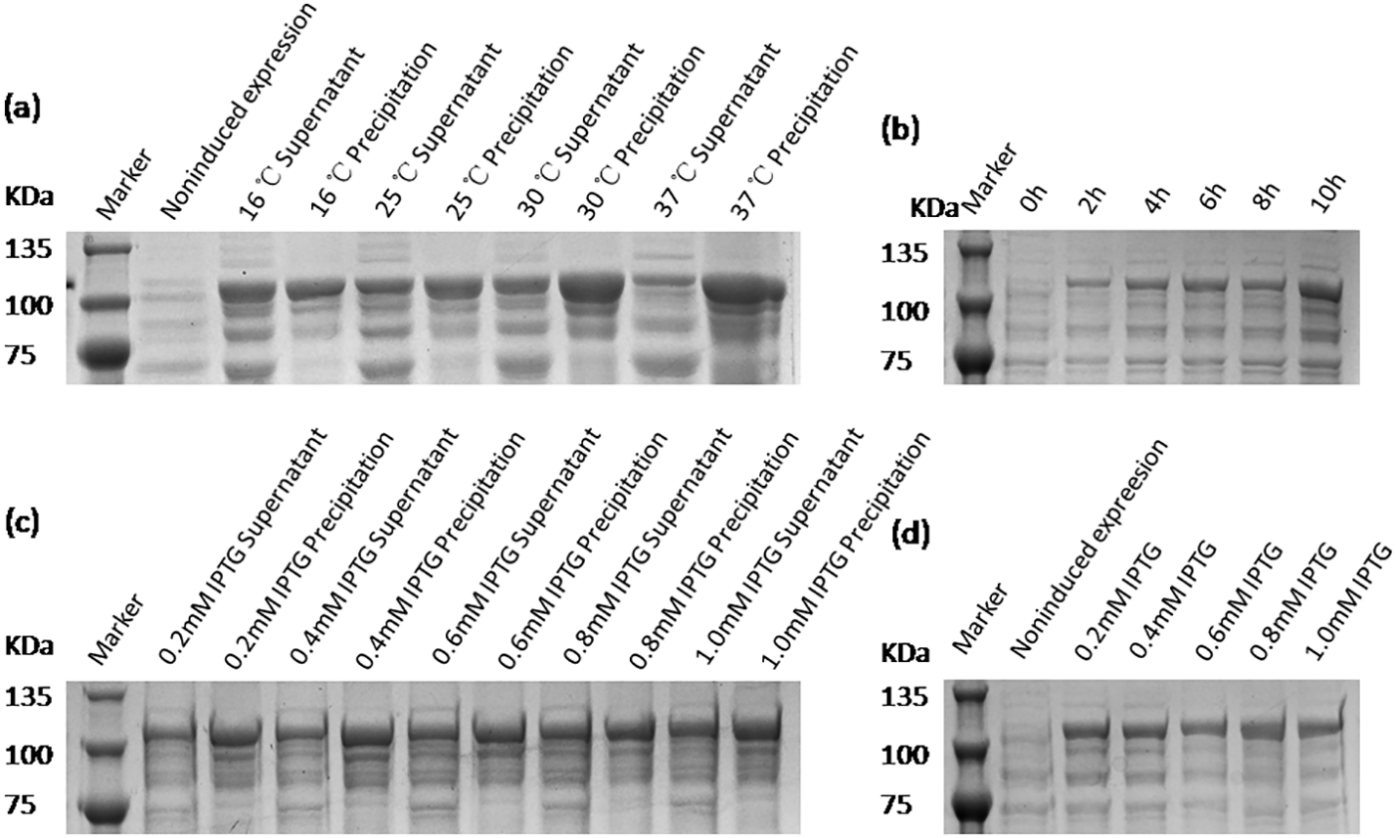
SDS-PAGE analysis of the optimal expression conditions of recombinant VP2 protein. Marker denotes the middle-ranged protein marker. (A) The optimization of inducing temperature. When the recombination strain was induced for 18 h by 0.5 mM IPTG at 16 °C, 25 °C, 30 °C, 20 µL of the culture supernatant was collected. The culture supernatant mixed with the 5 × loading buffer were boiled and then determined by SDS-PAGE. (B) The optimization of inducing time. When the recombination strain was induced by 0.5 mM IPTG at 16 °C from the beginning value 0 h to the ending value 10 h, 20 µL of the culture supernatant was collected and determined by SDS-PAGE. (C) and (D) The optimization of IPTG concentration. The fusion protein was induced for 18 h by a range of concentrations of IPTG containing 0.2 mmol/L, 0.4 mmol/L, 0.6 mmol/L, 0.8 mmol/L and 1.0 mmol/L for 18 h at 16 °C. The culture supernatant (D) was determined by SDS-PAGE. Then the induced cells were cracked by ultrasonic, the lysate supernatant and the lysate pellet (C) were analyzed by SDS-PAGE.

The expression conditions of recombinant protein VP2 were optimized in inducing temperatures, inducing times, and IPTG concentrations. As shown in Fig. 4, fusion protein attains its maximum expression quantity when induced by 0.8 mM IPTG at 16 °C for 10 h. The previous study has shown that excessively high IPTG concentrations will inhibit the growth of bacteria (Donovan, Robinson & Glick, 1996), so 0.4 mM IPTG was finally selected as the induced concentration.

### Purification of recombinant protein VP2

**Figure 5:**
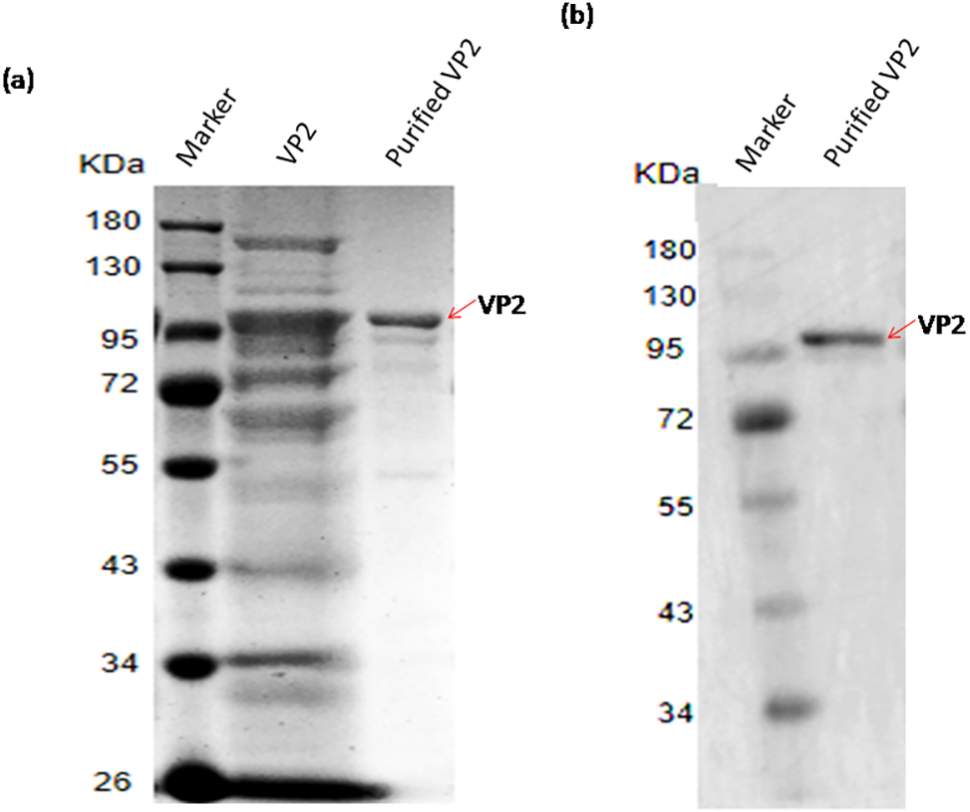
SDS-PAGE and Western blotting analysis of the purified recombinant VP2. Marker denotes the middle-ranged protein marker. (A) The induced cells were cracked by ultrasonic, the lyate supernatant was purified by the Ni-NTA agarose (Qiagen, Hilden, Germany), the purified VP2 was determined shown in lane 3 with the unpurified (lane 2) as control by SDS-PAGE. (B) Western blotting analysis of the purified VP2 by His-tag monoclonal antibody is shown in lane 2.

The recombinant protein VP2 was purified using Ni-chelating affinity chromatography. The purity of the target protein was above 90% explained by SDS-PAGE gel scan analysis (Fig. 5A) and western blotting (Fig. 5B). The concentration of VP2 was 0.2 g/mL determined by Bradford Protein Assay Kit and a total f 60 mg recombinant protein VP2 was obtained from 300 ml bacterial liquid.

#### Mass spectrometric identification of VP2 protein

**Figure 6:**
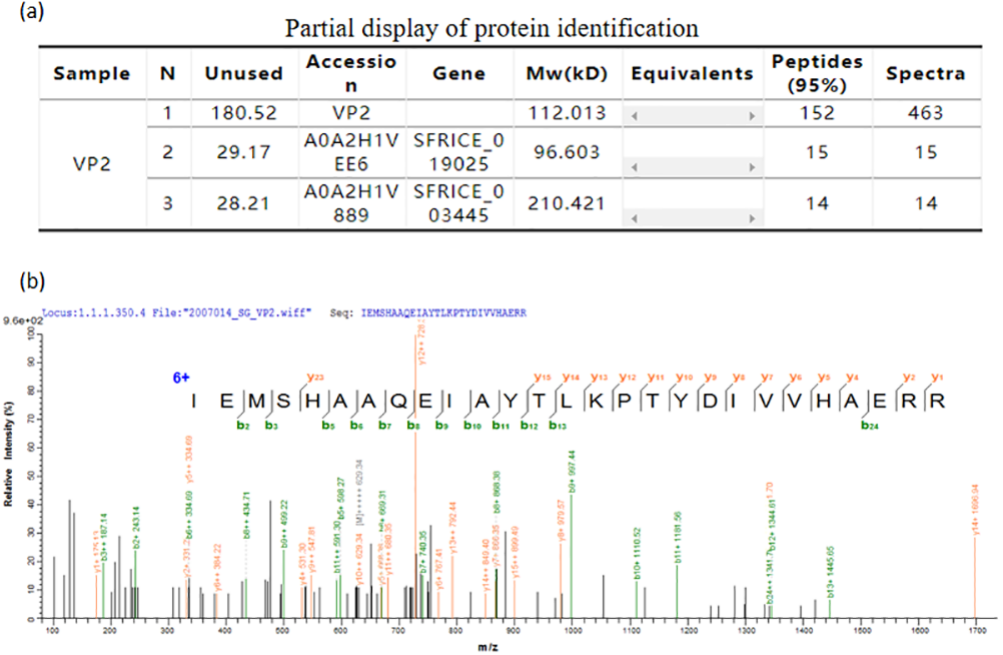
Mass spectrometric identification of VP2 protein. (A) Partial display of protein identification. (B) One of the mass spectra of trypsin digestion in the gel band.

The mass spectrometry identification result table (Fig. 6A), and one of the mass spectra of trypsin digestion in the gel band (Fig. 6B) showed that the peptide fragments of the protein we purified have a high degree of match with the VP2 protein sequence in the protein database. The results above indubitably determined the identity of the recombinant His-tagged VP2 protein.

### Reactivity of recombinant protein VP2 with BTV1 positive serum

**Figure 7:**
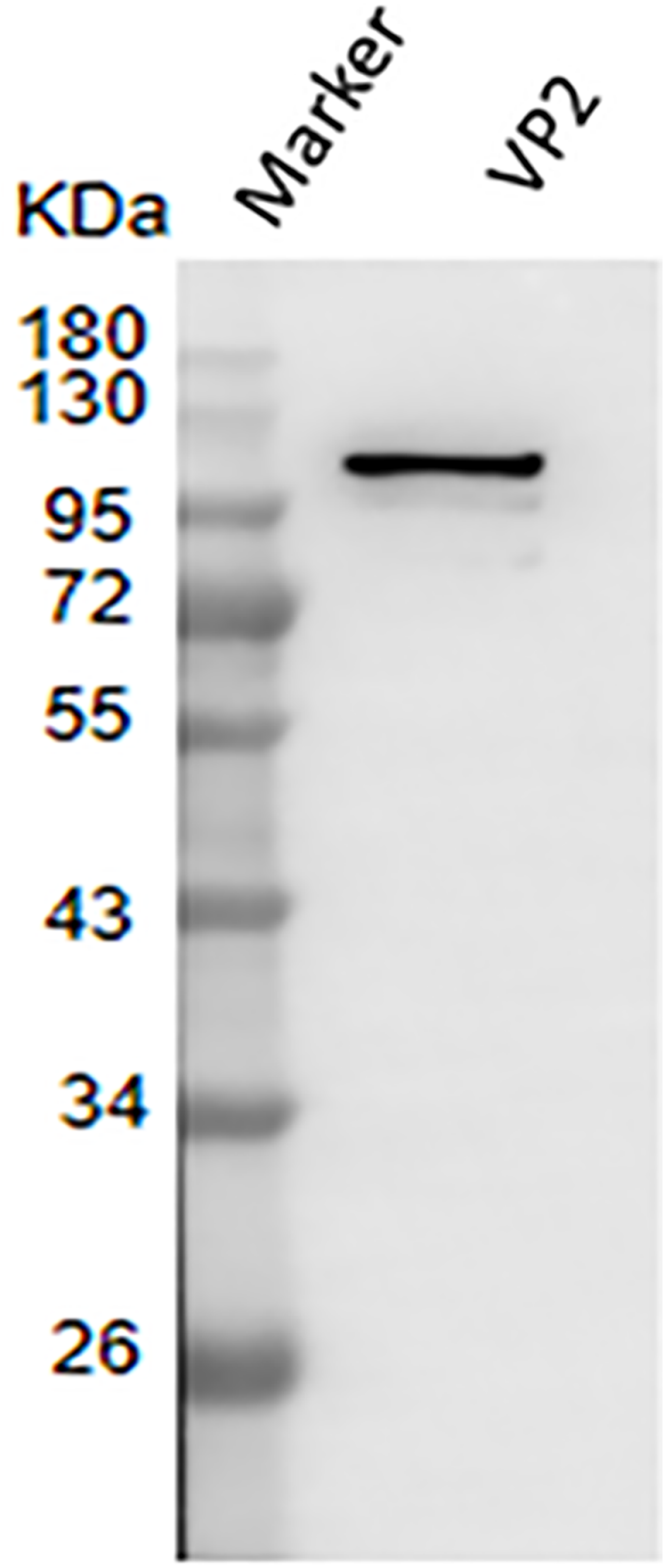
Western blotting to identify the immunoreactivity of recombinant protein VP2. Marker denotes the middle-ranged protein marker. Western blotting analysis of the VP2 protein by BTV1 positive serum demonstrated that a specific reaction band was ∼109 kDa in lane 2.

The result of western blotting showed that VP2 had a high reactivity with BTV1 positive serum (Fig. 7).

### Evaluation of immunization effect

**Figure 8:**
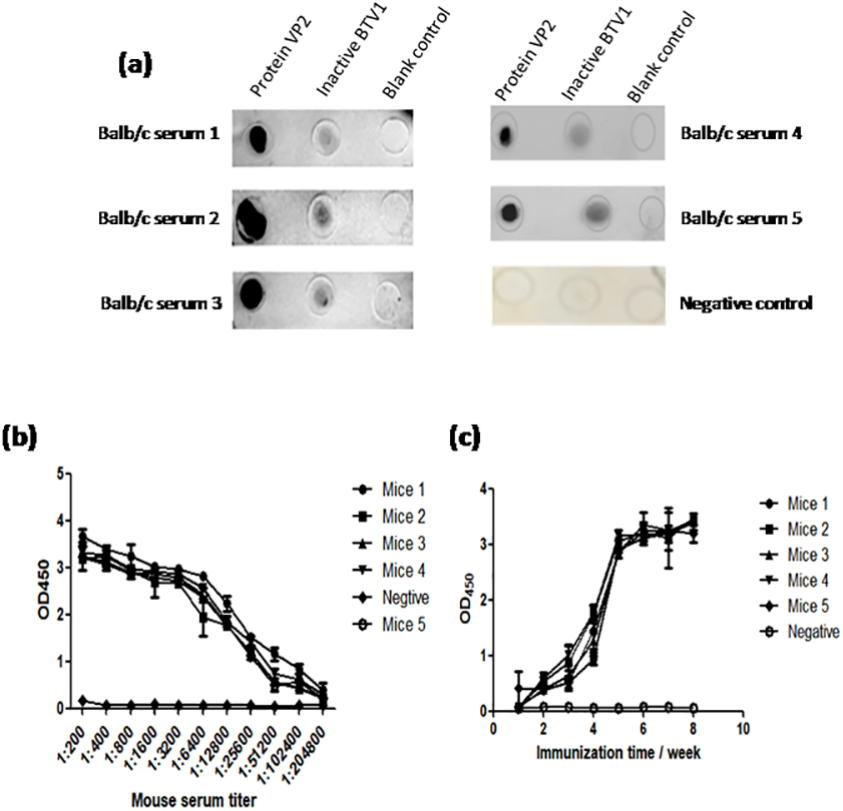
The immune effect of fusion protein VP2 was analyzed by dot-ELISA and indirect ELISA assay. The nitrocellulose filter membrane was coated by recombinant protein VP2 and inactivated BTV1. The sera of mice and HRP-conjugated goat anti-mouse IgG antibody were used as the primary antibody and the secondary antibody, respectively. Dot-ELISA analysis is shown (A); the microplate was coated with recombinant protein VP2 and primary antibodies was a series of gradient diluted mouse serum (mouse serum collected after the third immunization ) as well as the secondary antibody was HRP-conjugated goat anti-mouse IgG antibody. The serum titer with VP2 as coating protein is shown (B); the microplate was coated with 50 µl purified recombinant protein VP2 and primary antibodies was a series of serum collected from the first week to the eighth week after immunization, as well as the secondary antibody was HRP-conjugated goat anti-mouse IgG antibody. Anti-VP2 antibody titer changes with immunization time (C).

The results of dot-ELISA showed that the serum of mice immunized with recombinant protein VP2 could specifically react with BTV1 inactivated virus, while the serum of mice in the control group had no response (Fig. 8A). The serum titer of VP2 when used as coating protein was up to 1: 2.048 ×10 5 (Fig. 8B), and anti-VP2 antibody titer increased with immunization time (Fig. 8C).

## Discussion

Bluetongue virus causes a severe infectious disease BT. BTV has many serotypes and there is no cross-protection among different serotypes. It is difficult to control and eliminate BT using conventional methods due to its asymptomatic infection. Vaccination is a very effective way to control BT outbreaks (Baetza, 2014). However, the existing modified live vaccines and inactivated vaccines have some disadvantages. The modified live vaccine is high cost and its toxic residual may cripple ruminants (Savini et al., 2014; Savini et al., 2007). The inactivated vaccines need to immunize animals several times (González et al., 2010). Moreover, the nonreplicative vaccine is expensive and less effective and there are some problems in terms of safety and market acceptance for effective and cheap replication-vaccine (Feenstra & Van Rijn, 2016). All these efforts have been made with VLPs expressed in the baculovirus system for more than 20 years. Although VLPs vaccines have been achieved very promising results in large animal experiments, they have not yet been marketed (Stewart et al., 2010). The precise reason for current status has not yet been described in the literature, it may because that the production method is not cost-effective. Therefore, subunit vaccines will be a good choice to address these issues.

BTV structural protein VP2 can induce a protective immune response in sheep (Nunes et al., 2014). Moreover, it is not feasible to obtain protein VP2 using large-scale chemical purification (Huismans, Walt & Erasmus, 1985). It was reported that VP2 was expressed in the baculovirus expression system (Delos et al., 1993; Inumaru & Roy, 1987; Roy, French & Erasmus, 1992; Urakawa et al., 1994); two domains of VP2 were successfully expressed in the prokaryotic system (Mohd Jaafar et al., 2014), but the full-lengh recombinant protein of BTV1 VP2 was not expressed in prokaryotic expression system. The prokaryotic expression system is a mature expression system, which has the advantages of simple operation, easy purification, and mass production (Zhang et al., 2015). In this study, it is the first time to express the full-length BTV1 VP2 using the prokaryotic expression system. The recombinant BTV1 VP2 with good solubility reacts with BTV1 positive serum from mouse, and the immunization analysis showed that serum of mice immunized with VP2 displayed specifical reactivity with BTV1 inactivated virus. In addition, the immunized mice produced high-potency specific antibodies against VP2 protein. Sera from Balb/c mice immunised with the soluble recombinant VP2D1 of BTV-4, neutralised the homologous virus, while signifificantly lower NAb titres were observed with sera of mice immunised with soluble VP2D2 (Mohd Jaafar et al., 2014). These indicated that the recombinant protein VP2 we obtained has good Immunoreactivity.

Futhermore, the study also found that the reactivity of VP2 to BTV1 positive serum from mouse was weaker than that of VP7. It may be caused by the following reasons: VP2 is located in the outermost layer of the virion, which makes VP2 have highly hydrophilic and contains serotype-specific epitopes that may be recognized by neutralized hemagglutination inhibitor (HI) antibodies (Zhang et al., 2010); Amino acid sequence of VP2 is highly variable resulting in mutations amino acid cause the conformation changes of the VP2 protein and the spatial position changes of epitopes (Maan Narender et al., 2012); Another reason is that VP7 located on the outer surface of the core particles and contains BTV group-specific epitopes, which can stimulate the body to produce a strong group-specific immune response (Hosamani et al., 2011; Anthony et al., 2007).

## Conclusions

In this study, the recombinant vector pET28a-VP2 was successfully constructed, and the soluble recombinant VP2 was successfully obtained using the prokaryotic expression system. After optimizing the purification conditions, the target protein with high purity was finally obtained, which could specifically react with BTV1 positive serum. The recombinant VP2 was used to immunize Balb/c mice and the serum titer of indirect ELISA was 1: 2.048 ×10^5^. Dot-ELISA results showed that the serum of mice immunized with VP2 could react specifically with BTV1 inactivated virus.

In short, the recombinant protein BTV1 VP2 in the full-length sequence has been expressed for the first time in the Prokaryotic expression system. It does not only lay the foundation for the study of the structure and function of BTV1 VP2 but also the production of subunit vaccines provides a better theoretical proof.

##  Supplemental Information

10.7717/peerj.10543/supp-1 Supplemental Information 1 Figure and serum titer raw data

10.7717/peerj.10543/supp-2 Supplemental Information 2 Raw data exported from Mass spectrometric and Size Exclusion Chromatography Used in data analyses and preparation for Fig. 6.

10.7717/peerj.10543/supp-3 Supplemental Information 3 Mouse serum titer

10.7717/peerj.10543/supp-4 Supplemental Information 4 Regularity of antibody growth Scatter plot of anti-VP2 antibody titer increased with immunization time (Fig. 8C).

10.7717/peerj.10543/supp-5 Supplemental Information 5 Serum titer data

10.7717/peerj.10543/supp-6 Supplemental Information 6 Regularity of antibody growth data Raw data of anti-VP2 antibody titer after 1 8 weeks of immunization exported from the Microplate Reader (BioTek Instruments, Inc, Winooski, VT, US) for data analyses and preparation for Fig. 8C.

10.7717/peerj.10543/supp-7 Supplemental Information 7 VP2/5/7/inactivated BTV1 with BTV1 positive serum

## Additional Information and Declarations

### Data Availability

The following information was supplied regarding data availability: The raw measurements are available in the Supplemental Files.
