# Supplementary figures and images for: Soluble expression and purification of Bluetongue Virus Type 1 (BTV1) structure protein VP2 in Escherichia coli and its immunogenicity in mice
Source: PeerJ. 2021 Jan 4;9:e10543. doi: 10.7717/peerj.10543 (PMC7789859; doi:10.7717/peerj.10543)

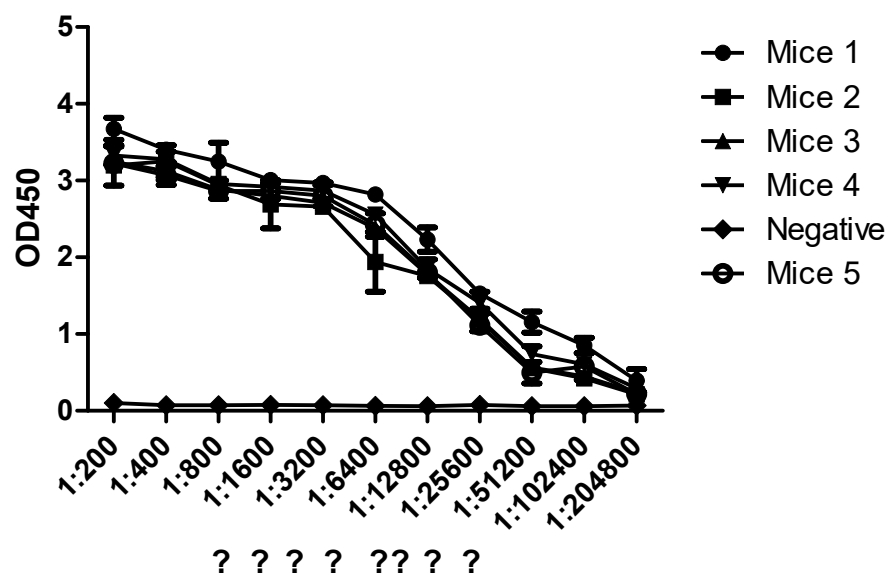

Supplement: Supplemental Information 3 [file peerj-09-10543-s003.pdf]

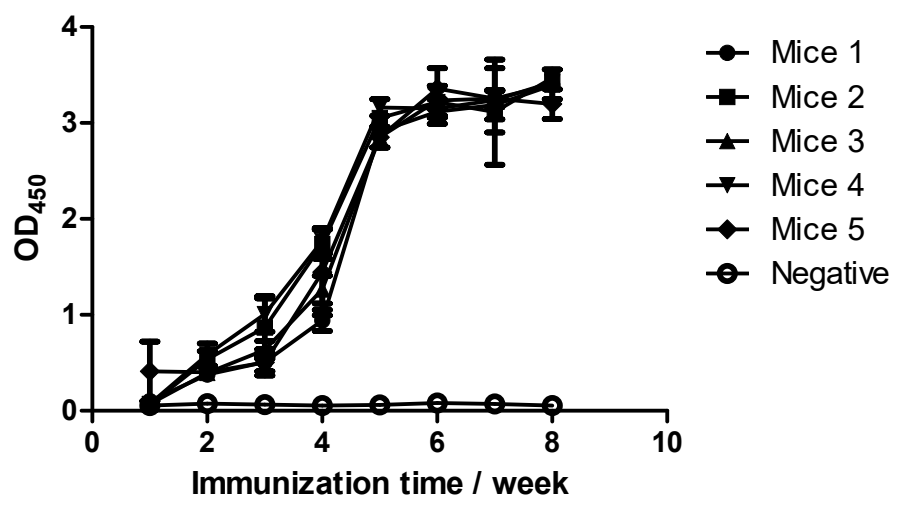

Supplement: Supplemental Information 4 — Scatter plot of anti-VP2 antibody titer increased with immunization time (Fig. 8C). [file peerj-09-10543-s004.pdf]
