# Supplementary material for: Soluble expression and purification of Bluetongue Virus Type 1 (BTV1) structure protein VP2 in Escherichia coli and its immunogenicity in mice
Source: PeerJ. 2021 Jan 4;9:e10543. doi: 10.7717/peerj.10543 (PMC7789859; doi:10.7717/peerj.10543)
Supplement: Supplemental Information 7 [file peerj-09-10543-s007.docx]

The reaction results were compared：


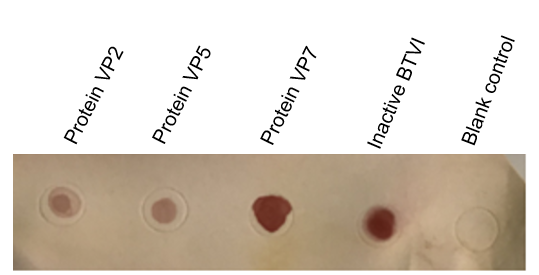


The result of the reaction of BTV1 VP2/VP5/VP7/inactivated BTV1 with BTV1 positive serum.
